# Progressive Left Ventricular Outflow Tract Stenosis in a Noonan Syndrome Patient With Severe Hypertrophic Cardiomyopathy During Growth Hormone Treatment

**DOI:** 10.7759/cureus.23937

**Published:** 2022-04-07

**Authors:** Yasuhiro Ichikawa, Naka Saito, Kenji Kurosawa, Junko Hanakawa, Hideaki Ueda

**Affiliations:** 1 Cardiology, Kanagawa Children's Medical Center, Yokohama, JPN; 2 Clinical Laboratory, Kanagawa Children's Medical Center, Yokohama, JPN; 3 Medical Genetics, Kanagawa Children's Medical Center, Yokohama, JPN; 4 Endocrinology and Metabolism, Kanagawa Children's Medical Center, Yokohama, JPN

**Keywords:** raf1, left ventricular outflow tract stenosis, hypertrophic cardiomyopathy, growth hormone, noonan syndrome

## Abstract

Short stature is a main problem in Noonan syndrome (NS). Recombinant human growth hormone (GH) has been used to safely improve the growth rate in NS patients with short stature. However, there is little information about GH therapy for NS associated with hypertrophic obstructive cardiomyopathy. We present the case of a seven-year-old NS patient with severe hypertrophic obstructive cardiomyopathy. The patient received GH therapy for six months, at which time progressive left ventricular outflow tract stenosis was apparent.

## Introduction

Noonan syndrome (NS) is an autosomal dominant disorder that is characterized by short stature, craniofacial dysmorphism, congenital heart disease, skeletal abnormalities, developmental delay, hematologic disorder, and other abnormalities [1]. The most common cardiovascular problems in NS are pulmonary valve stenosis, hypertrophic cardiomyopathy (HCM), and atrial septal defect.

Short stature affects 50%-70% of patients with NS and is one of its main diagnostic features [2]. Patients with NS receive growth hormone (GH) therapy to treat short stature [3,4]. One of the main concerns about GH treatment is the presence of HCM because patients with acromegaly and a chronic excess of GH and insulin-like growth factor-1 (IGF-1) exhibit biventricular hypertrophy [2,5]. In multiple clinical trials that included patients with HCM, there was no evidence to support a higher prevalence of cardiac comorbidities, and GH treatment was shown to be safe [2,4]. However, there is insufficient information about GH therapy in NS associated with HCM [6].

We report the case of a seven-year-old male NS patient with severe hypertrophic obstructive cardiomyopathy (HOCM) who received GH therapy that resulted in progressive left ventricular outflow tract stenosis (LVOTS).

## Case presentation

A seven-year-old male was born full term and was the first child. Because thickening of the left ventricular wall was observed in utero, he was admitted to the neonatal intensive care unit after birth. An echocardiogram showed HCM without left ventricular outflow tract stenosis. At two months of age, the patient was discharged and started on diuretics and propranolol with careful cardiac follow-up.

On the basis of the patient’s clinical findings, NS was suspected. Genetic testing revealed a heterozygous missense variant on exon 7, c.770C>T (p.S257L) in RAF1. Genetic testing of the patient’s parents found that no mutations were present. Thus, the NS diagnosis was confirmed.

Despite receiving medication, the echocardiogram showed that the LVOTS had worsened, and the patient exhibited HOCM. Diagnostic cardiac catheterization was performed and showed a pressure gradient from the left ventricular apex to the ascending aorta that was 60 mmHg at two years of age. When the patient was three years old, septal myotomy-myectomy was performed to treat the LVOTS. After surgery, peak velocity of the left ventricular outflow tract was improved and mitral regurgitation was mild.

**Figure 1:**
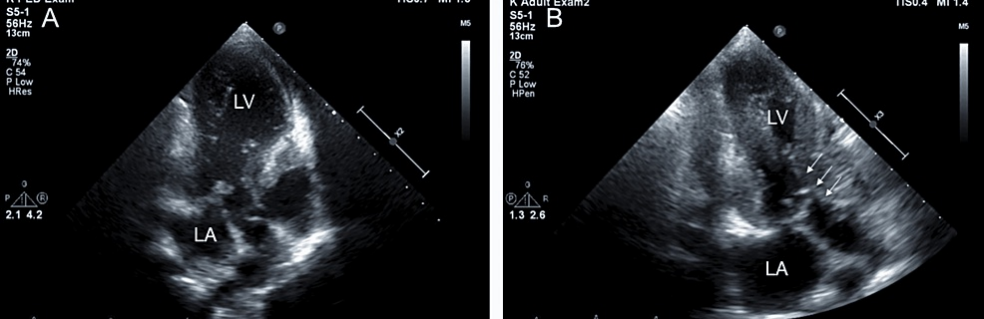
Echocardiogram results for our patient focused on left ventricular outflow tract stenosis. (A) Before GH treatment. (B) Six months after the patient started GH treatment. GH: growth hormone, LV: left ventricle, LA: left atrium Arrows indicate left ventricular outflow tract stenosis.

**Figure 2:**
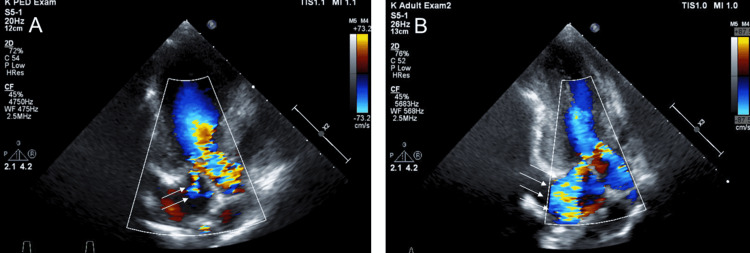
Echocardiogram results for our patient focused on mitral regurgitation. (A) Before GH treatment. (B) Six months after the patient started GH treatment. GH: growth hormone Arrows indicate progressive mitral regurgitation.

When the patient was four years and six months old, patient’s height and weight were 87.7 cm (−3.85 standard deviation [SD]) and 13.35 kg (−1.75 SD), respectively. GH treatment was started at the guardian’s request despite severe HCM. Before GH treatment, he was referred to the pediatric cardiologist. The echocardiogram results showed left ventricle dimension diastole of 23.5 mm, ejection fraction of 74.7% and left ventricle (LV) wall thickness at diastolic phase of 8.5 mm, mild mitral regurgitation, and mild LVOTS (peak velocity of the left ventricular outflow tract was 2.9 m/s) (Figures 1A, 2A). A GH stimulation test revealed GH deficiency (GHD) (Table 1). Table 2 shows the patient’s laboratory data before GH treatment. White blood cell fraction results were negative for juvenile myelomonocytic leukemia. Based on these results, the GH dose at the start of therapy was 0.088 mg/kg/week (half the dose for GHD).

**Table 1:** Growth hormone stimulation test GH: Growth hormone, GHRP-2: Growth Hormone Releasing Peptide-2

		0 min	15 min	30 min	45 min	60 min
GHRP-2 test	GH (ng/mL)	2.51	6.45	11.66	10.96	7.62

**Table 2:** Laboratory data before growth hormone treatment TSH: thyroid stimulating hormone, IGF-1: insulin-like growth factor 1

Test Components	
white blood cell count	16100 /μL
red blood cell count	505×10^4/μL
hemoglobin	13.2 g/dL
platelets	42.7×10^4/μL
TSH	2.92 μIU/mL
fT3 (free triiodothyronine)	5.07 pg/mL
fT4 (free thyroxine)	1.70 ng/dL
IGF-1	47 ng/mL

Cardiac follow-up was performed during GH treatment. When the patient was five years old, a follow-up echocardiogram showed progressive LVOTS (peak velocity of left ventricular outflow tract was 5.0 m/s) and progressive mitral regurgitation due to systolic anterior movement (Figures 1B, 2B). Although the patient was re-prescribed propranolol to treat LVOTS, echocardiogram results showed preserved left ventricular contraction (left ventricle dimension diastole of 30.6 mm, ejection fraction of 59.8%), increased LV wall thickness (LV wall thickness at diastolic phase of 11.4 mm), and left ventricular outflow tract velocity worsened (peak left ventricular outflow tract velocity was 5.8 m/s) at five years and seven months of age. GH treatment was stopped due to progressive LVOTS.

The patient is now seven years old, and his mitral regurgitation and LVOTS have not worsened.

## Discussion

We present the case of a NS patient with severe HCM who underwent septal myotomy-myectomy. The patient received GH treatment for short stature, and after starting GH, LVOTS progressed.

Several clinical trial results indicated that GH therapy is safe for as long as cardiac follow-up is performed during GH therapy. Some clinical trials have included patients with NS complicated by HCM [4]. In a previous report, the effects of growth hormone on the left ventricular wall in patients with NS, including one patient with HOCM, were studied for five years, but the left ventricular wall thickness did not change [3,4].

In our case, the patient exhibited a severe HCM phenotype due to RAF1 gene mutations, and low-dose GH was administered [7]. Because the number of cases is small and HOCM severity is unclear in previous reports, information about GH treatment for severe HCM was limited [2]. For NS patients with severe HCM, current knowledge is insufficient to conclude if GH treatment is safe in patients with NS and pre-existing severe HCM. The cause of LVOTS progression in our patient is unclear, but it may have occurred after GH treatment was started, and GH may have had an effect on LVOTS. Further careful study is warranted to examine GH therapy side effects in NS patients with severe HCM. 

## Conclusions

GH treatment is a safe and effective therapy for NS patients for short stature. However, GH therapy for patients with severe HCM is not well understood. Cardiologists should be mindful that careful use of GH therapy will be required in patients with NS and pre-existing severe HCM. Frequent cardiac follow-up during GH therapy is also necessary.

## References

[1] Roberts AE, Allanson JE, Tartaglia M, Gelb BD (2013). Noonan syndrome. Lancet.

[2] Edouard T, Zenker M, Östman-Smith I (2022). Management of growth failure and other endocrine aspects in patients with Noonan syndrome across Europe: a sub-analysis of a European clinical practice survey. Eur J Med Genet.

[3] Cotterill AM, McKenna WJ, Brady AF (1996). The short-term effects of growth hormone therapy on height velocity and cardiac ventricular wall thickness in children with Noonan’s syndrome. J Clin Endocrinol Metab.

[4] Rohrer TR, Abuzzahab J, Backeljauw P (2021). Long-term effectiveness and safety of childhood growth hormone treatment in Noonan syndrome. Horm Res Paediatr.

[5] Clayton RN (2003). Cardiovascular function in acromegaly. Endocr Rev.

[6] Romano AA, Dana K, Bakker B, Davis DA, Hunold JJ, Jacobs J, Lippe B (2009). Growth response, near-adult height, and patterns of growth and puberty in patients with Noonan syndrome treated with growth hormone. J Clin Endocrinol Metab.

[7] Pandit B, Sarkozy A, Pennacchio LA (2007). Gain-of-function RAF1 mutations cause Noonan and LEOPARD syndromes with hypertrophic cardiomyopathy. Nat Genet.

